# Molecular and biochemical characterization of rice developed through conventional integration of *nDart1-0* transposon gene

**DOI:** 10.1038/s41598-023-35095-7

**Published:** 2023-05-19

**Authors:** Sanaullah Jalil, Qurban Ali, Asad Ullah Khan, Muhammad Mudassir Nazir, Sharafat Ali, Faisal Zulfiqar, Muhammad Arshad Javed, Xiaoli Jin

**Affiliations:** 1 Department of Agronomy, Zhejiang University, Hangzhou, 310058 Zhejiang China; 2 Crop Sciences Institute, National Agricultural Research Center, Islamabad, 44000 Pakistan; 3 Department of Plant Breeding and Genetics, Faculty of Agricultural Sciences, University of the Punjab, Lahore, 54590 Pakistan; 4 Department of Horticultural Sciences, Faculty of Agriculture and Environment, The Islamia University of Bahawalpur, Bahawalpur, 63100 Pakistan

**Keywords:** Biotechnology, Plant biotechnology, Molecular engineering in plants

## Abstract

Mutations, the genetic variations in genomic sequences, play an important role in molecular biology and biotechnology. During DNA replication or meiosis, one of the mutations is transposons or jumping genes. An indigenous transposon *nDart1-0* was successfully introduced into local indica cultivar Basmati-370 from transposon-tagged line viz., GR-7895 (japonica genotype) through conventional breeding technique, successive backcrossing. Plants from segregating populationsshowed variegated phenotypes were tagged as *BM-37* mutants. Blast analysis of the sequence data revealed that the GTP-binding protein, located on the BAC clone OJ1781_H11 of chromosome 5, contained an insertion of DNA transposon *nDart1-0*. The *nDart1-0* has “A” at position 254 bp, whereas *nDart1* homologs have “G”, which efficiently distinguishes *nDart1-0* from its homologs. The histological analysis revealed that the chloroplast of mesophyll cells in *BM-37* was disrupted with reduction in size of starch granules and higher number of osmophillic plastoglobuli, which resulted in decreased chlorophyll contents and carotenoids, gas exchange parameters (*Pn, g, E, Ci*), and reduced expression level of genes associated with chlorophyll biosynthesis, photosynthesis and chloroplast development. Along with the rise of GTP protein, the salicylic acid (SA) and gibberellic acid (GA) and antioxidant contents(SOD) and MDA levels significantly enhanced, while, the cytokinins (CK), ascorbate peroxidase (APX), catalase (CAT), total flavanoid contents (TFC) and total phenolic contents (TPC) significantly reduced in BM-37 mutant plants as compared with WT plants. These results support the notion that GTP-binding proteins influence the process underlying chloroplast formation. Therefore, it is anticipated that to combat biotic or abiotic stress conditions, the *nDart1-0* tagged mutant (*BM-37*) of Basmati-370 would be beneficial.

## Introduction

A monocot semi-aquatic annual grass plant, rice is a member of the Poaceae family and belongs to the genus *Oryza*^1^. The Oryza genus has 22 wild taxa, of which two species are highly significant for human consumption^2^. These species are *Oryza sativa* L., generally known as Asian rice, and *Oryza glaberrima* Steud, popularly known as African rice. Several classifications within the Oryza genus include *Oryza officinalis*, *Oryza ridelyi*, and *Oryza rufipogon*. Of the 22 wild species of *Oryza*, 15 originated in Asia and 7 in sub-Saharan Africa^3^. These species are beneficial in interspecific rice breeding programs because wild *Oryza* species have particular traits, including abiotic and biotic stress tolerance^4,5^. Over half of all humans eat rice, making it the most important cereal crop^6^.

Moreover, the full genome sequence of rice has been sequenced with great fidelity, and comparatively high number of transposons were found, making it a great model for the study of transposons^7,8^. An *nDart1-0* transposon (*pyl-v*) was found in a rice virescent rice mutant with light yellow variegated leaves of Taichung-65 (*Oryza sativa* japonica L.)^9^. In particular, genotypes, containing *aDart1-27* an active autonomous DNA element, carry a transposon gene, the element *nDart1* are actively transferred throughout the genome^10^. The *nDart1* elements, which belong to the superfamily *hAT*, are excised from chromosomal inserted locations and transposed to other sites, despite having a high degree of sequence similarity, *nDart1-0* and the closely comparable non-autonomous components *nDart1-1* through *nDart1-12* display differing transposition frequencies^11^. Active transposons are frequently employed to tag genes and reveal their functionalities^12,13^. A tagging method is an effective tool, because nDart1 elements are actively transposed into genome and have a propensity to integrate at genomic locations. The nDart1 elements, especially transposed into locations at the proximal promoter of the genome, which are located 0.5 kb before the putative initiation codons^14^. The iPCR-based methods and transposon display (TD) methods were also created to efficiently locate inserted sites of nDart1 with in genome^15,16^.

Both the *japonica* and *indica* subspecies of rice have had their whole genomes sequenced, however the experimental research on many functional genes in the rice genome is still ongoing^17^. Examining how many genes work is now one of the most difficult goals. According to the descriptions of certain specific genes, numerous intermediate components of developmental processes, namely proteins involved in signal transduction pathways, are still substantially conserved. The G protein is a fantastic illustration of these so-called molecular switches^18^. Through intrinsic activities, these proteins undergo conformational changes brought on by the binding and hydrolysis of GTP^14^. GTP-binding proteins differ in eukaryotes and prokaryotes and are among the most important proteins for all species^19^. GTPases or G-proteins are other names for these proteins. It is a molecular switch that is present in all areas of life. It is “activated” by the GTP molecule and “inactivated” by the hydrolysis of the GTP molecule to GDP. It regulates several cellular functions, including the rearrangement of the cell cytoskeleton, signal transduction, translation, transcriptional regulation, vesicle trafficking, and protein transport^20,21^. The G1, G2, G3, G4, and G5 motifs are present in all G-proteins. These substances are necessary for GTP hydrolysis, GTP conformational change, and GDP/GTP conversion. In the G-protein family, G2 pattern is robustly maintained but has little impact on GDP/GTP exchange activity^22,23^. The aim of this study was to introduce transposon *nDart1-0* into highly growing local *Indica* rice cultivar “Basmati-370” to obtain mutant through conventional breeding approach and to characterize the ERA-like GTP-binding protein gene and its effects on the activation of phytohormones, which may be helpful to induce biotic/ abiotic stress tolerance in rice.

## Materials and methods

### Plant materials

The required breeding material for the current experiment was collected from two different sources; seed of *Indica* rice cultivar, Basmati-370 from Rice Research Institute, Kala Shah Kaku, Punjab, Pakistan, and seed of japonica line GR-7895 (consisting *nDart1-0* transposon) from Plant Genetic Resources Institute, National Agricultural Research Center (NARC), Islamabad, Pakistan. It has been confirmed that the experimental data collection complied with relevant institutional, national, and international guidelines and legislation with appropriate permissions from Plant Genetic Resources Institute, National Agricultural Research Center (NARC), Islamabad, Pakistan. The data on search experiments revealed with the studies on Basmati-370 have shown that it does not carry any of the aDart elements. The aDart is actually a carrier for *nDart1-0* transposon element. For study purposes, mutants were developed for gene tagging by using insertion of the transposon aDart element. A cross was carried out between Basmati-370 and GR-7895 for insertion of aDart element on Basmati-370 from GR-7895 genotype. Dry mature seeds from both lines were surface sterilized for this purpose and raised in controlled environments in pots. To synchronize flowering for crossover, Basmati-370 seeds were cultivated at one-week intervals for four successive weeks. The crossing was carried out by keeping Basmati-370 as a female parent. The F_1_ seeds were grown under controlled conditions and backcrossed with designated female plants of Basmati-370 to produce BC_1_F_1_ and continue this backcrossing to produce BC_4_F_1_ (Supplementary material: Fig. S1). The harvested seeds from BC_4_F_1_ were grown and self-pollinated to get BC_4_F_2_. The seeds from BC_4_F_2_ were harvested and further grown under controlled conditions to get segregating population upto BC_4_F_4_ of plants to identify the abnormal or albino phenotypes used for gene tagging.

### Isolation and quantification of DNA from the transposon-induced mutants

The DNA was extracted from the mutant plants through using a protocol of DNA isolation given by Doyle^24^. The extracted DNA was then transferred into eppendorf; along with added 7 μl of RNAase, which incubated at 37 ℃ for 60 min. The quality of DNA was verified using 2% agarose gels, while its quantity was measured using Nanodrop. The samples were then diluted for uniform and standard concentration of DNA up to 1 μl of each sample per 50 µl dH_2_O. The isolated DNA was then quantified by taking a reading of absorbance at 260 nm wavelength keeping using dH_2_O as a blank sample.

### RNA extraction and qRT-PCR analysis

RNA was isolated from leaves of rice plants from Basmati-370 (WT) and *BM-37* (mutant) through the TRIeasyTM Total RNA Extraction Reagent Kit (Yeasen Biotechnology Co., Ltd. Shanghai, China) as per the manufacturer's guidelines. The quality of RNA was verified using 2% agarose gels, while its quantity was measured using Nanodrop. The cDNA was synthesized by an improved qRT-PCR technique derived from the total mRNA^25,26^. PCR was performed for amplifing of full length cDNA of GTP using specific primers. Oligonucleotide primers (Supplementary material: Table S1) for the amplification of full-length cDNA of OJ1781_H11 were designed based on the reported sequence in rice (Accession No. AC120986). PCR amplified sequence was cloned and sequenced. The sequence comparison and data analysis were performed using blast with the NCBI database.

We examined the expression level of *nDart1-0* in Basmati-370 (*BM-37*) mutant plants, including plumules, third and fifth leaves at the seedlings stage, roots, stems, sheath, young panicles, and flag leaves at the heading stage of plants. Quantitative RT-PCR tests were conducted to measure the amount of nDart1 expression. An additional set of genes (*PORA, CAO1, Cab1R, YGL1, CHLD, HEMA, PsbA, RNRS, RbcL, PsaA, RbcS, LhcpII, OsPoLP1, RNRL, FtsZ, Rpl21, RpoB, Rsp20, RpoA, OsRpoTp* and *Rps7* related of photosynthesis, chloroplast development and chlorophyll biosynthesis were examined for their expression level through qRT-PCR. Primers designed for qRT-PCR are mentioned in Table S2 (Supplementary material). The conditions for gene amplifications are stated in (Supplementary material: Fig. S2).

### Analysis of the mutants through n1-0SPiPCR

The genomic DNA from leaves of *BM-37* mutant plants was isolated according toDellaporta et al.^27^. All the mutants were analyzed by n1-0SPiPCR method (Supplementary material; File 1). For PCR, the following conditions were adjusted: an activation phase at 95 °C for 30 s, denaturation with 35 cycles at 60℃ for 30 s, annealing at 72 ℃ for 60 s, and extension at 72 ℃ for 10 min. By designing primers through software application Primer_premier5 from the gene's flanking area around the transposon's insertion site, the transposon fragments were amplified.

### TEM analysis

The leaf samples from Basmati-370 (WT) and mutant (*BM-37*) plants devoid of veins were taken from randomly selected seedlings and placed in 2.5% glutaraldehyde in 0.1 M phosphate buffer with pH 7.0 for 5 h, then washed three times with phosphate buffer (0.1 M, pH 7.0). Further, the samples were postfixed with 1% OsO4 for 1 h and washed three times with phosphate buffer (0.1 M, pH 7.0) for 15 min at each wash. Then, samples were dehydrated with graded series of ethanol (30, 50. 70, 80, 90 and 100%, respectively) and doused with concentrated acetone for 20 min, and ultimately imbedded in Spurr's medium before being cut into ultrathin sections. Moreover, the samples after cutting in ultra-thin sections were placed on the copper nets for observation under transmission electron microscope (JEOLTEM-1230EX).

### Measurement of levels for chlorophyll pigments, carotenoids and gas exchange parameters

Utilizing a spectrophotometer by making minor adjustments in the methods of Arnon and Wellburn^28,29^, the carotenoid (Car), total chlorophyll, and chlorophyll (a and b) were assessed. In a nutshell, 0.2 g samples from leaves of seedlings grown under controlled circumstances at the three-leaf stage were collected and homogenized for 18 h in the dark in 5 ml of ethanol:water:acetone (4:1:5) solution. Centrifugation was used to get rid of leftover particles. A UV5100 Spectrophotometer was used to evaluate the supernatants. In addition, the portable photosynthetic system LI-6400 was used to quantify the transpiration rate (*E*), the concentration of intracellular CO_2_ (*Ci*), stomatal conductance (*g*), and net photosynthetic rate (*Pn*).

### Determination of endogenous phytohormones

The total phenolic contents were calculated using the Folin-Ciocalteu method, with gallic acid acting as a controlled chemical^30^. Equivalents of gallic acid per gram of FW-fresh weight were used to indicate the amount of total phenolic compounds. By employing catechin as a reference ingredient, the aluminum trichloride technique was used to measure the flavonoids concentration^31^. (gE catechin.100 g1 FW) was used to express the overall flavonoid content. Following the manufacturer's recommendations, we used a plant ELISA kit to detect the endogenous salicylic acid (SA), abscisic acid (ABA), gibberellic acid (GA) and cytokinins (CK). To measure the concentrations of SA, ABA, GA and CK in the samples, leaves were pulverized in liquid nitrogen. The hormonal SA, ABA, GA and CK ELISA kits come with a set of calibration standards. The SA, ABA, GA and CK levels in the samples were then measured by fitting the standard curve and generating a comparable trend in the samples.

### Measurement of antioxidants, H_2_O_2_ and malondialdehyde (MDA) contents

The leaf samples were homogenized in sodium phosphate buffer (pH 7.8), centrifuged at 13,000 rpm for 20 min at 4 °C. Then superoxide dismutase (SOD) activity was measured spectrophotometrically as described by Zhang et al.^32^, at 560 nm by assessing the ability of each unit to inhibit 50% photochemical reduction of nitro blue tetrazolium chloride (NBT). Peroxidase (POD) activity was determined according to Zhou and Leul^33^, at 470 nm and the changes related to guaiacol were normalized with the activity constant (ε = 26.6 mm) and catalase (CAT) activity was determined according to Aebi^34^. The APX activity was calculated depending on the decrease in absorbance at 290 nm according to Nakano and Asada^35^ as ascorbate was oxidized. This study measured reactive oxygen species in plant tissues, including hydrogen peroxide (H_2_O_2_). Hydrogen peroxide content was measured as described by Velikova et al.^36^. Malondialdehyde (MDA) content was determined according to Morales and Munné-Bosch,^37^. The total phenolic content was measured by the Folin Ciocalteu method using gallic acid as a reference compound^30^. The flavonoids content was determined by the aluminum trichloride method using catechin as reference compound^38^.

### Statistical analysis

The standard error and mean from three replications are reported for each set of data. Data analysis was carried out using Statistics 8.1, a statistical software programme. The recorded data was analyzed through one way variance analysis and LSD test and Tukey’s test to determine pairwise significance at *p *˂ 0.05. Origin Pro (version 8.5) was used to create the graphs.

## Results

To study gene action, the regulation and characterization of genes have become the most important genetic tool in biotechnology and genetic engineering due to reverse and forward genetic approaches. For the evaluation of genetic potential and variations in the population of crop plants, there is necessary to develop mutants by using irradiations, chemicals, transposons, and induced genetic mutations for tagging genes to carry out functional genomic evaluations of species genomes from narrow to more comprehensive ranges. In our current study, we have described a active, and non-autonomous genomic or DNA transposable element in rice, named non-autonomous DNA-based active rice transposon one (*nDart1*). The *nDart1* is a causative transposable element of allele with spontaneous mutable and virescent nature, the pale-yellow-leaf variegated or *pyl-v*, which caused pale yellow with dark-green streaks on leaves of rice plants at very early seedling stages usually due to the expression of *nDart1* gene in rice. There are multiple transposon elements along with their multiple gene insertional sites, which a crop plant line of genotype has been found in limited numbers because of the reduced requirements for the identification and characterization of specific genes tagging a population genome for achieving maximum saturation of transposable elements. For our current study, we have selected the nDart1 gene to produce rice mutants, which were expressed as pale-yellow-leaf variegated. The *nDart1* gene was inserted into the Basmit-370 variety through the conventional backcross breeding method.

### Development and phenotypic appearance of mutants

**Figure 1:**
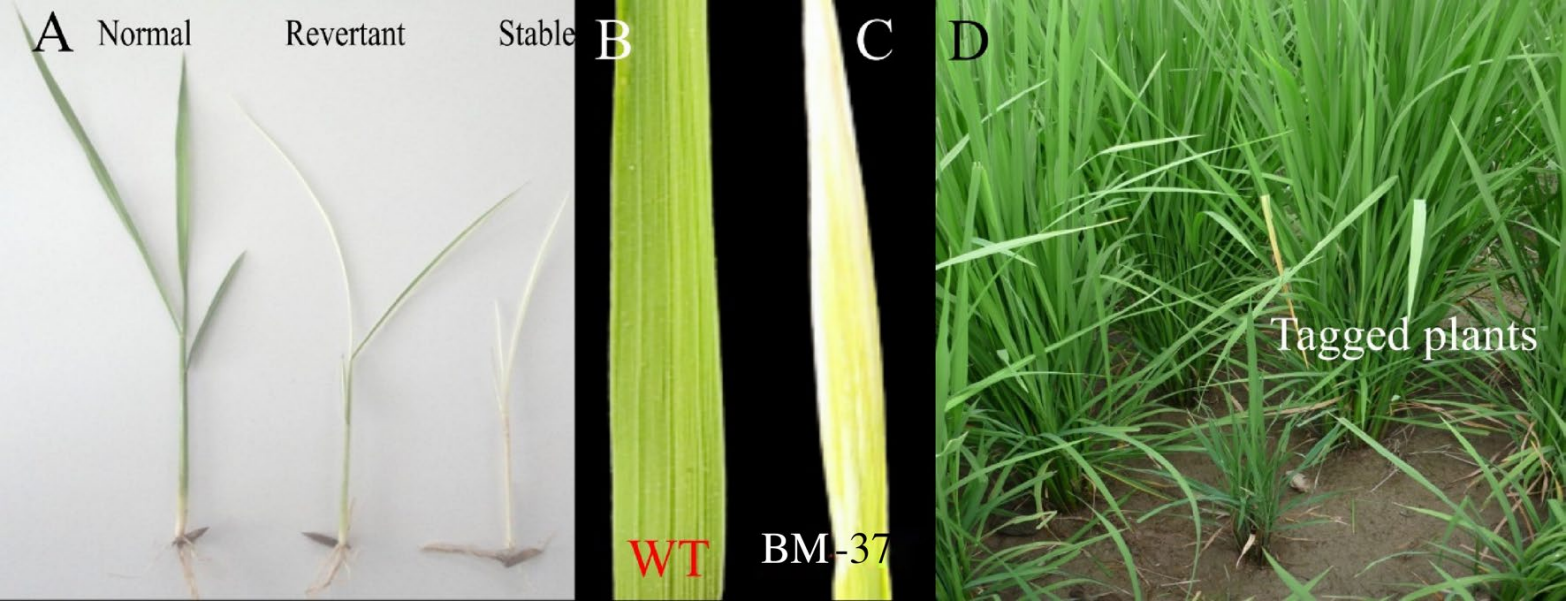
Transposon-induced mutants from segregating population. (**A**) Phenotypic difference between normal, revertant, and stable albino seedlings; (**C**, **D**) phenotypic appearance of third leaves at the seedling stage from WT and *BM-37* mutant; (**D**) segregated mutant plants for tagging of genes.

In our current research study, we have utilized an endogenous and available transposon named *nDart1* to develop mutants for tagging genes in the Basmati-370 rice genotype. As we could not find any active nDarts or active autonomous element Darts in local basmati varieties, we introduced japonica-derived *aDart* and *nDart1-0* into the *indica* rice variety Basmati-370 through backcrossing for mutant genes. Different plants were selected from segregating populations that showed variation and were tagged as mutants (Fig. 1A) from the BC_4_F_4_ population. Through genetic analysis, this mutant was surmised to be controlled by a two-component transposon system.

During growing of rice plants in the field after BC_4_F_4,_ there were normal green leaves in the WT and variegated types of albino rice plants in *BM-37* (Fig. 1B, C), which was introduced with nDart1/aDart1. The lines which showed gene expression were postulated to have a recessive allele that had the *nDart1-0* homozygous as well as an active autonomous element as aDart1 with heterozygous allele conditions. It was found that the mutated line produced stable albino progenies (Fig. 1D). The stable mutants indicated that there were not only stable GTP homozygous alleles but also having without an aDart1element with a ratio of 3:1 for F_2_. It was also found that there was no difference between the WT as well as the mutants in rice seeds, but the difference was found only at the stage of seedling growth, where the normal seeds produced normal green leaves and stem, whereas the mutated seeds produced white shoots and leaves. The results indicated the presence of *aDart1* elements as found stable in rice plant phenotypes. It was found that aDart1-dependent *nDart1-0* somatic excisions of allele produced a leaf with variegations that developed throughout cell lineage or progenies of mutated individuals of rice plant. However, the white mutated or variegated plants may grow their panicles with fertile grains under extreme conditions.

### Gene tagging by nDart1-0-iPCR method

The frequency of spontaneous mutation under natural growth conditions is extremely low. Transposons are useful for gene tagging and functional analysis to create natural variation, so it is useful to explore active transposon sources. Mutant phenotypes were tagged in the field and DNA was isolated from phenotypically variegated leaves of mutants. DNA was subjected to restriction digestions and self-ligation. Data search revealed that nDart1-0 has thirteen homologs and a high homology above 99%. At position 254 bp, nDart1-0 has “A”, whereas the homologs have “G” as shown in Table S3 (Supplementary material). The site “A” was used efficiently to distinguish nDart1-0 from its homologs.

**Figure 2:**
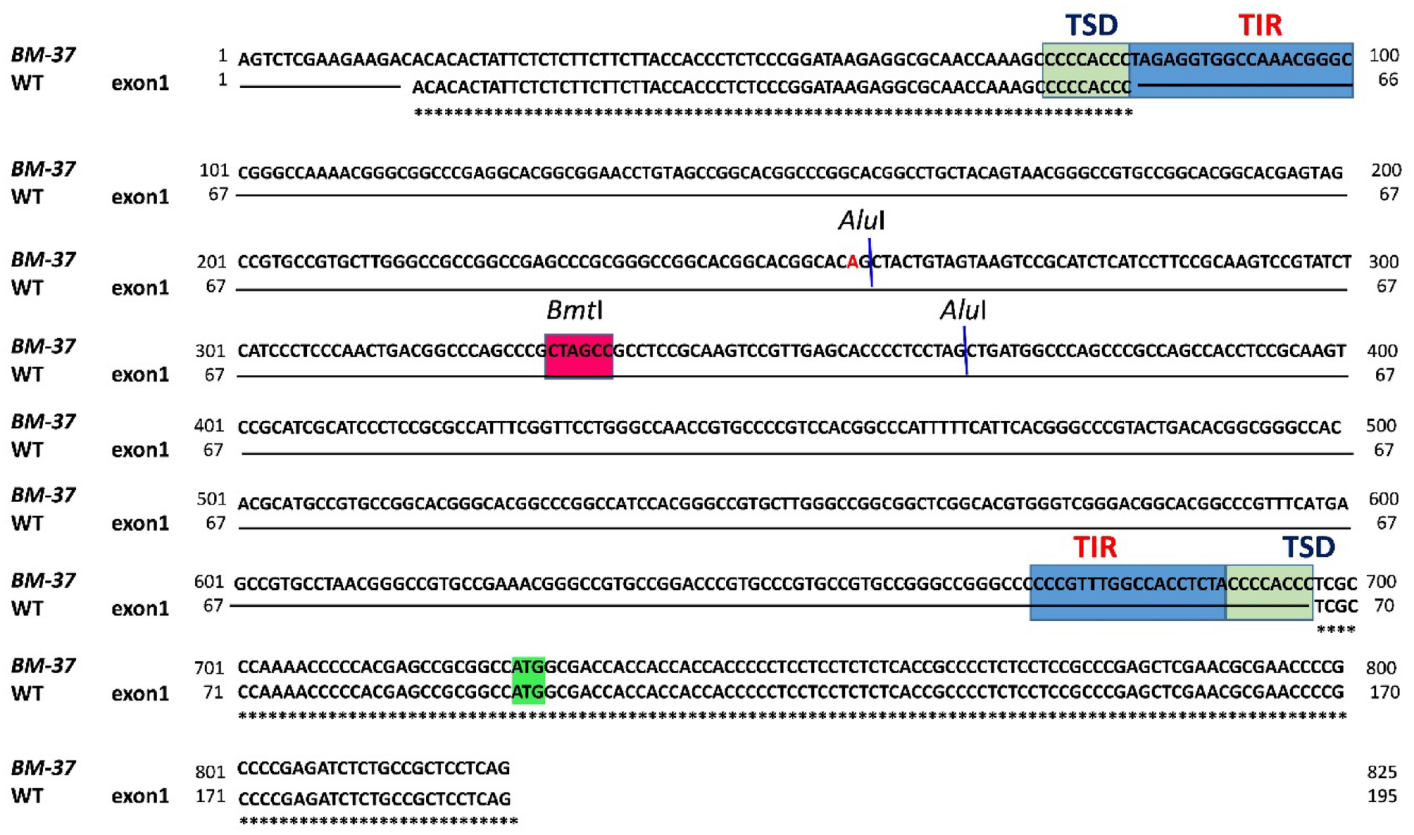
The *nDart1-0* transposon and its homologues in rice. A Sequence of *nDart1-0* transposon in rice. Arrows show primer positionArrows and restriction sites show primer positions are highlighted in red. TSD-target site duplication; TIRs-terminal inverted repeats.

Keeping in view that according to this technique, DNA is restricted with *Alu* I, self-ligated, and again subject to another restriction digestion with *Bmt* I and amplification by iPCR using specific primers. Digestion by *Alu* I, restrict *nDart1-0* at position 254 as it carries a unique specific restriction site due to the substitution of a base pair “A” whereas homologs carry “G” at this site during self-ligation, this digested fragment of the *nDart1-0* ligates with a fragment of the gene invaded by him (Fig. 2). Self-ligation yields some specific fragments having a part of the *nDart1-0* and the gene invaded by him along with other fragments. Again, restriction of this self-ligated DNA by *Bmt* I cleaves the *nDart1-0* as it carries *Bmt* I site, as a result, we get some fragment of the invaded gene flanked by *nDart1-0*. These fragments were amplified through iPCR using the specific primers designed for flanking region of *nDart1-0*. The two rounds or cycles of iPCR (like pre-selective and selective gene amplifications) were carried out for confirmation of results. During the selective type of amplification, the further enhancement and enrichment of transposon elements or their related specific fragments were achieved through using available PCR master mix products like a template to carry out the second transposable element amplification with the nested type of primer, which specifically recognized highly conserved transposable element regions in the genome. Nonspecific genomic rearrangements during iPCR are ruled out because of the primers derived from specific regions of the *nDart1-0*. On the other hand, the nDart homologues does not carry *Alu* I site at position 254, they are not digested at this position and consequently are not amplified with specific primers during iPCR (Supplementary material: Fig. S3A). When DNA restricted with *Alu* I and self-ligated is used as template for iPCR, nDart1-0 and all its homologues are amplified but this amplification is restricted to nDart1-0 if self-ligated DNA is again restricted with *Bmt* I. For confirmation of fragments obtained either these were transposable elements or not detected transpose element fragments were amplified to study the transposon element flanking sequences, PCR amplified fragments were sequenced and analyzed. The amplification patterns for each fragment were similar i.e., flanked by *nDart1-0*, except for the difference in genes invaded. So far, we have detected 12 genic regions invaded by *nDart1-0* in *BM-37* mutant (Supplementary material: Table S3). It was found that there was a mutant amplified fragment which was containing *nDart1-0* flanking sequences were detected which were derived due to the insertion of *nDart1-0* on to the first exon regions of a putative gene (Supplementary material: Fig. S3B).

**Figure 3:**
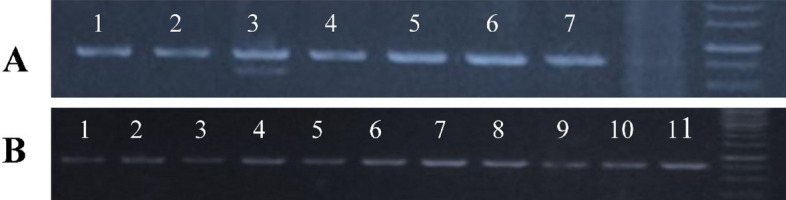
PCR amplification of nDart1-0 mutants and transcripts of GTP binding protein gene in several tissues of Basmati 370. (**A**) PCR amplification of *nDart1-0* mutants. Lane 1: Basmati 370, Lane2: T-65, Lane3: Nipponbare, Lane4: Mutable whitish-leaf, Lane 5: Stable whitish leaf-1, Lane6: Stable whitish leaf-2, Lane7: Stable whitish leaf-3; (**B**) transcripts of GTP binding protein gene in several tissues of Basmati 370. Lane 1: Etiolated plant leaf, Lane2: Etiloated plant root, Lane 3: 2-week-old plant leaf, Lane 4: 2-week-old plant root, Lane5: 2-Month plant leaf, Lane6: 2-Month old plant root, Lane 7: 2Month plant stem, Lane8: 2-Month old plant meristem, Lane9: 2-Month old plant glume, Lane 10: 2-Month old plant anther, Lane11-2Month old plant stigma.

The results from our study have suggested that the *nDart1-0* element insertion as well as its excision into the putative gene was as OJ1781_H11 on chromosome 5. Blast analysis of the sequence data revealed that GTP-binding protein locus on the BAC clone OJ1781_H11 of chromosome 5 was found to contain an insertion of DNA transposon *nDart1-0*. This gene has a nucleotide sequence of 2701 bp and 918 cDNA. The ORF encoded a polypeptide of 305 amino acid sequence and comprises 7 exons interrupted by 6 introns. During the process of gene tagging five revertants were found. These revertant phenotypes were analyzed to find out absence or presence of the transposon. DNA was isolated from the revertant phenotypes and transposon-inserted regions were amplified by the already-designed primers from the flanking regions of the transposon. Amplified fragments (Fig. 3A, B) were cloned and sequenced. Sequence analysis revealed that the transposon moved from the insertion point, leaving behind the footprints.

### Expression analysis of *BM-37* mutant

**Figure 4:**
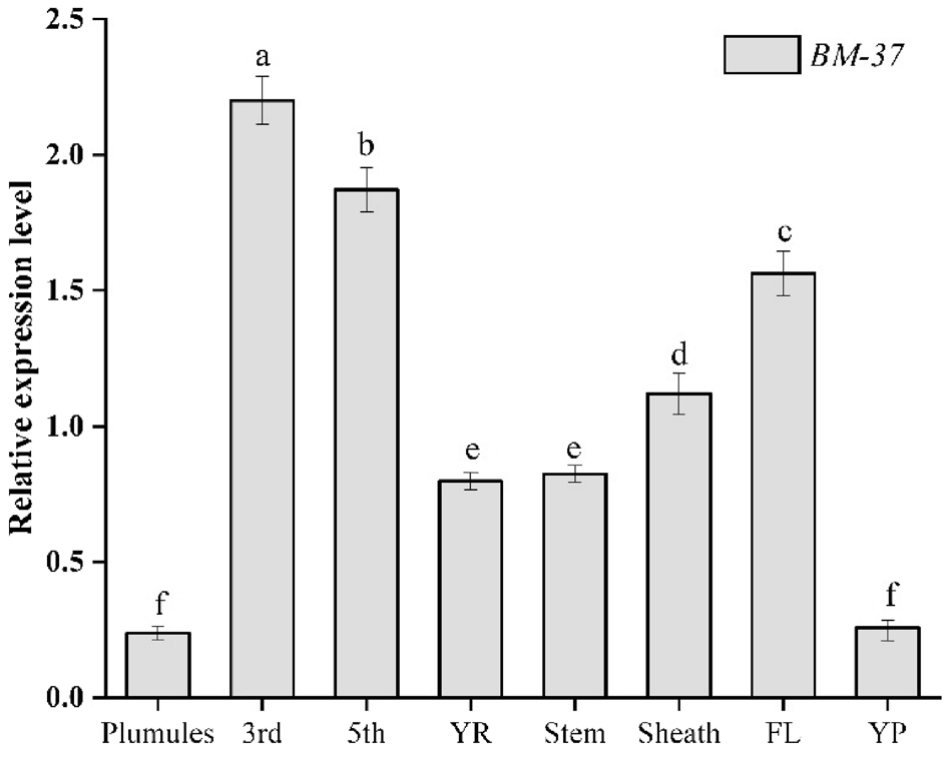
Transcript levels for expression study of different tissues in *BM-37* mutant. Plumules; 3rd-third leaves at the seedling stage; 5th-fifth leaves at the seedling stage; YR-young roots; Stem, Sheath, FL-flag leaves and YP-young panicle. All values represent the mean ± SD of five replicates. Different letters above error bars indicate the significant differences between treatments at p ≤ 0.05.

To study the expression pattern of transposon *nDart1-0*, the qRT-PCR was carried out on several tissue levels (3rd and 5th leaves at the seedling stage, plumules, sheath, shoots, roots, young panicles and flag leaves at heading stage)of *BM-37* mutant. The highest expression was found in the third leaves stage of seedling, compared to the fifth and flag leaves, across all the tissues analyzed, indicating that early seedling leaves had higher expression than other tissues. The tissues, including stem, sheath, and roots, expressed significantly lower expression level than leaves, while young panicles and plumules showed a minor level of expression for *BM-37* (Fig. 4). The findings suggested that *nDart1-0* have an influential role in leaf chloroplast development particularly during the early seedling stage.

### Ultrastructural analysis of mesophyll cells

**Figure 5:**
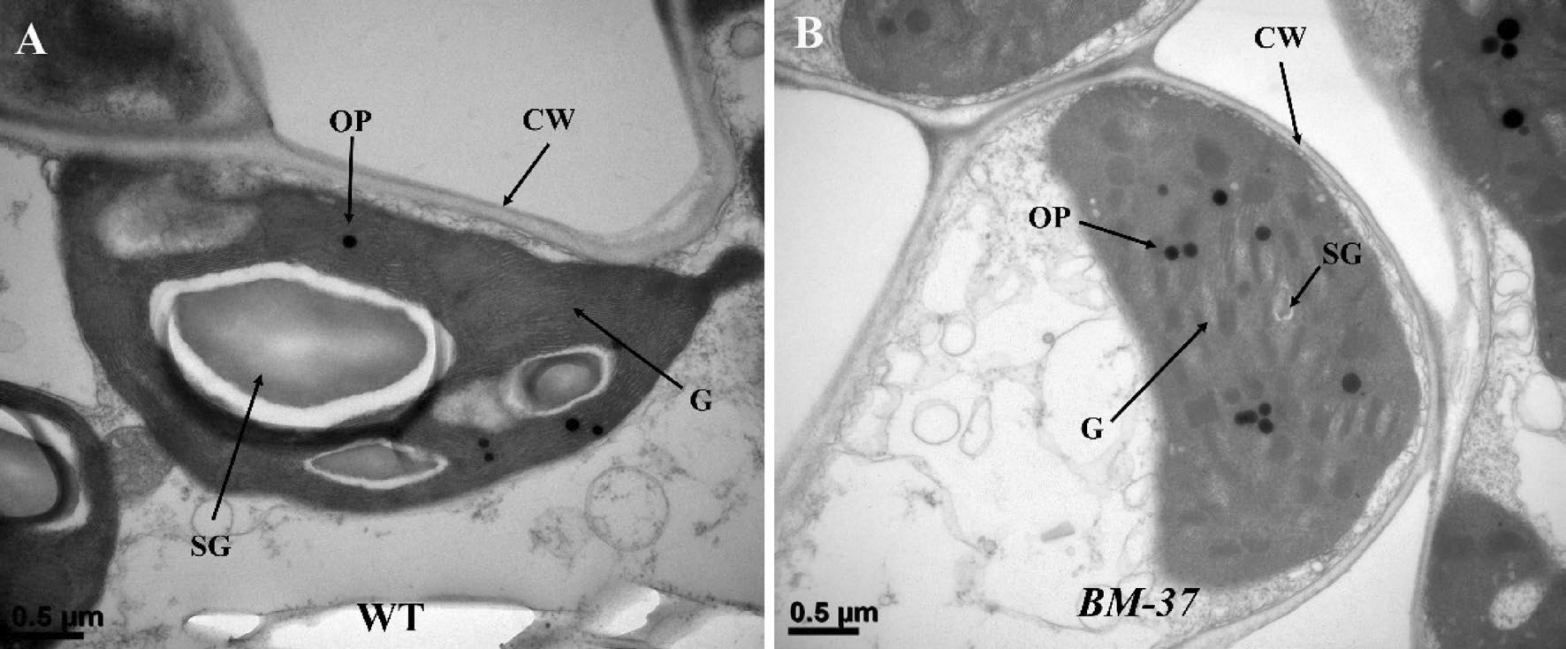
TEM images of chloroplasts in plants of WT and *BM-37* mutant (**A** and **B**). G, grana stacks; OP, osmophilic plastglobuli; SG, starch granules.

Using TEM, the upper part of leaves at the tillering stage were studied to examine the ultra-structural alterations in the chloroplast of WT and *BM-37* mutant plants. In contrast to the *BM-37* chloroplasts, which showed well-developed lamella structures, the grana lamella stacks in WT plants were sparse and sparsely populated. This suggests that the *BM-37* mutation had a significant impact on biogenesis. The outcome also demonstrated that starch granules were difficult to locate in the chloroplast of the *BM-37* mutant (Fig. 5A, B), although they were present normally in the WT. In the *BM-37* mutant, there were more osmophilic plastoglobuli (OP) than in the WT, indicating that the chlorophyll metabolism was substantially compromised.

### Transposon *n-Dart1-0* gene interrupted the pigment metabolism and photosynthetic efficiency

**Figure 6:**
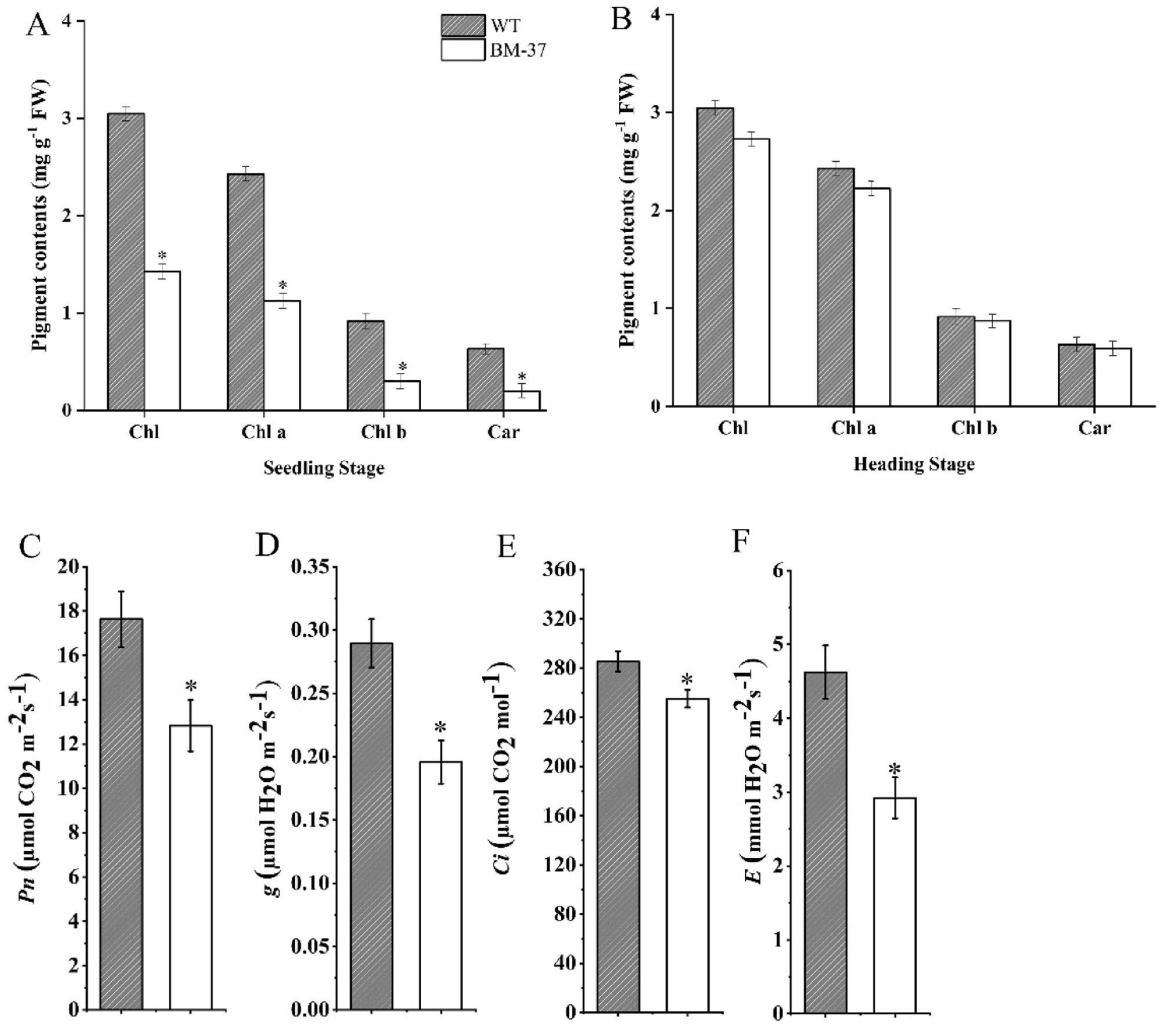
Chlorophyll pigment contents, carotenoid contents, and photosynthetic parameters analysis. (**A**) Chlorophyll and carotenoid contents in leaves at seedling stage of *BM-37* mutant and WT; (**B**) chlorophyll and carotenoid contents in leaves at heading stage of *BM-37* mutant and WT (**C**) net photosynthetic rate (*Pn*); (**D**) stomatal conductance (*g*); (**E**) intercellular CO_2_ concentration (Ci); (**F**) transpiration rate (*E*). All values represent the mean ± SD of five replicates. According to Tukey's test, asterisks indicate the statistical significance levels (*p < 0.05).

At the seedling and heading phases of the WT and *BM-37* plants, the carotenoid and chlorophyll contents were examined to ascertain the impact of the *nDart1-0* transposon on photosynthesis pigment metabolism. The chlorophyll (a, b and a + b) contents and carotenoid levels were drastically decreased in *BM-37* at seedling stage as compared with WT; however, the chlorophyll contents and carotenoids showed non-significant results at the heading stage in both WT and BM-37 mutant plants (Fig. 6A, B). Additionally, the gas exchange parameters were measured to compare the photosynthetic characteristics of WT and *BM-37* mutant plants. As compared to WT, the *BM-37* mutant dramatically lowered the rates of photosynthetic activity (*Pn*), stomatal conductance (*g*), intercellular CO_2_ concentration (*Ci*), and transpiration (*E*) (Fig. 6C–F).

### The *BM-37* regulates the expression of photosynthesis-associated genes

**Figure 7:**
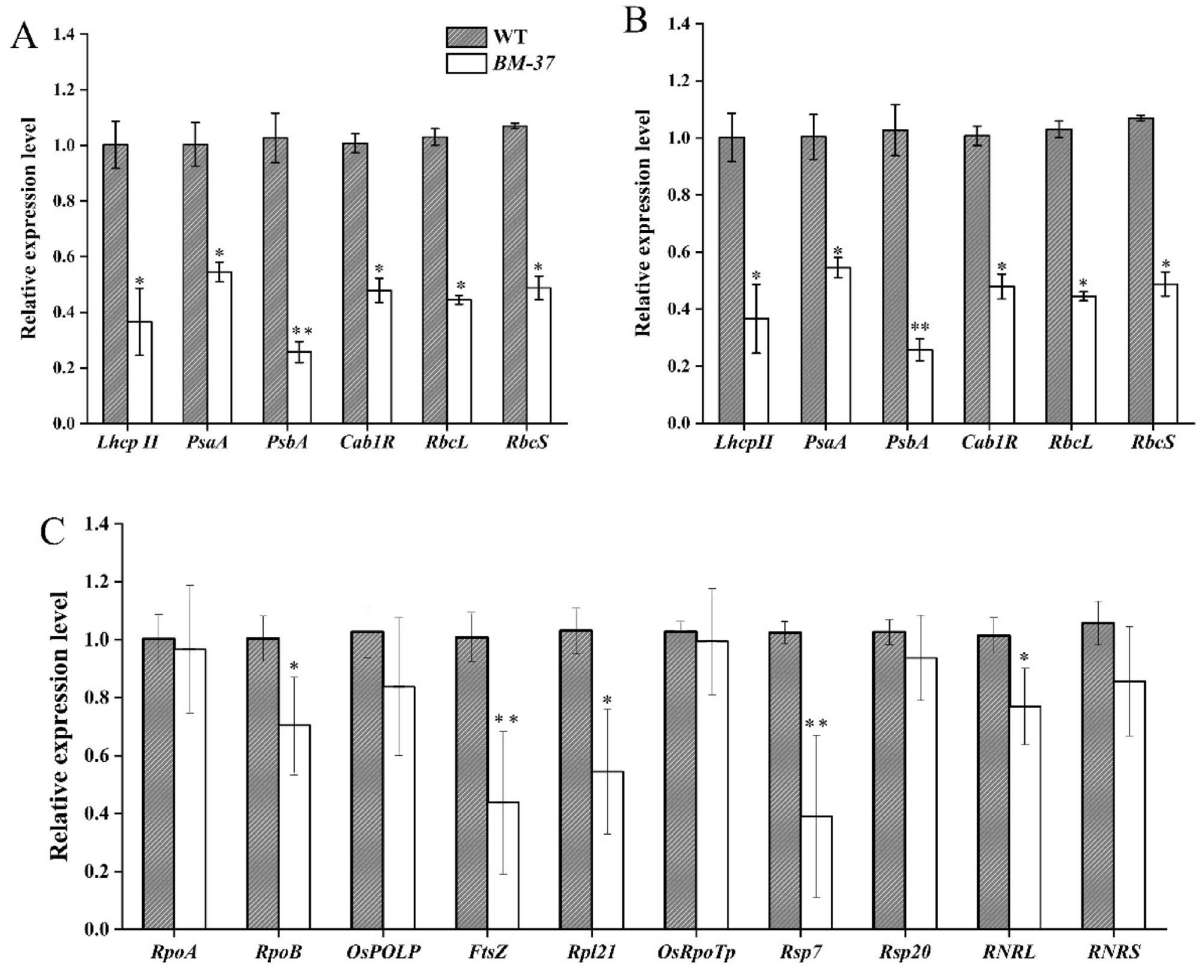
Quantitative expression analysis of genes related to (**A**) chlorophyll biosynthesis, (**B**) photosynthesis, and (**C**) chloroplast development in the *BM-37* mutant and WT plants.The expression level of each gene was analyzed by qPCR.

In WT plants and *BM-37* mutant plants, the expression levels for different genes related to chloroplast development, photosynthesis, and chlorophyll biosynthesis were evaluated at the seedling stage. The results showed that genes associated with biosynthesis of chlorophyll; comprising *PORA, CHLD, YGLI, CAO1* and *HEMA* and the photosynthesis related genes such as *RbcS, Cab1R, PsaA, PsbA, RbcL* and *LhcpII*^39,40^, were significantly down regulated in *pyl-v37* mutant plants than the WT plants (Fig. 7A, B).

We also studied the expression level of different genes related to chloroplast development, including *RpoA, RpoB, OsRpoTp, Rps7, Rps20, RNRL, RNRS, OsPolP1, FtsZ* and *Rpl21*^41–46^. Relative expression levels of all genes associated with chloroplast development were significantly down regulated in the *BM-37* mutant as comparable to WT plants (Fig. 7C). Under abiotic stress, it's possible that these important genes' aberrant expression caused the mutant phenotype. The findings showed that the *BM-37* mutation had a significant impact on the metabolism of chlorophyll, photosynthesis and chloroplasts development in *BM-37* mutant cells.

### Ultra-structural alterations in chloroplast caused oxidative damage

**Figure 8:**
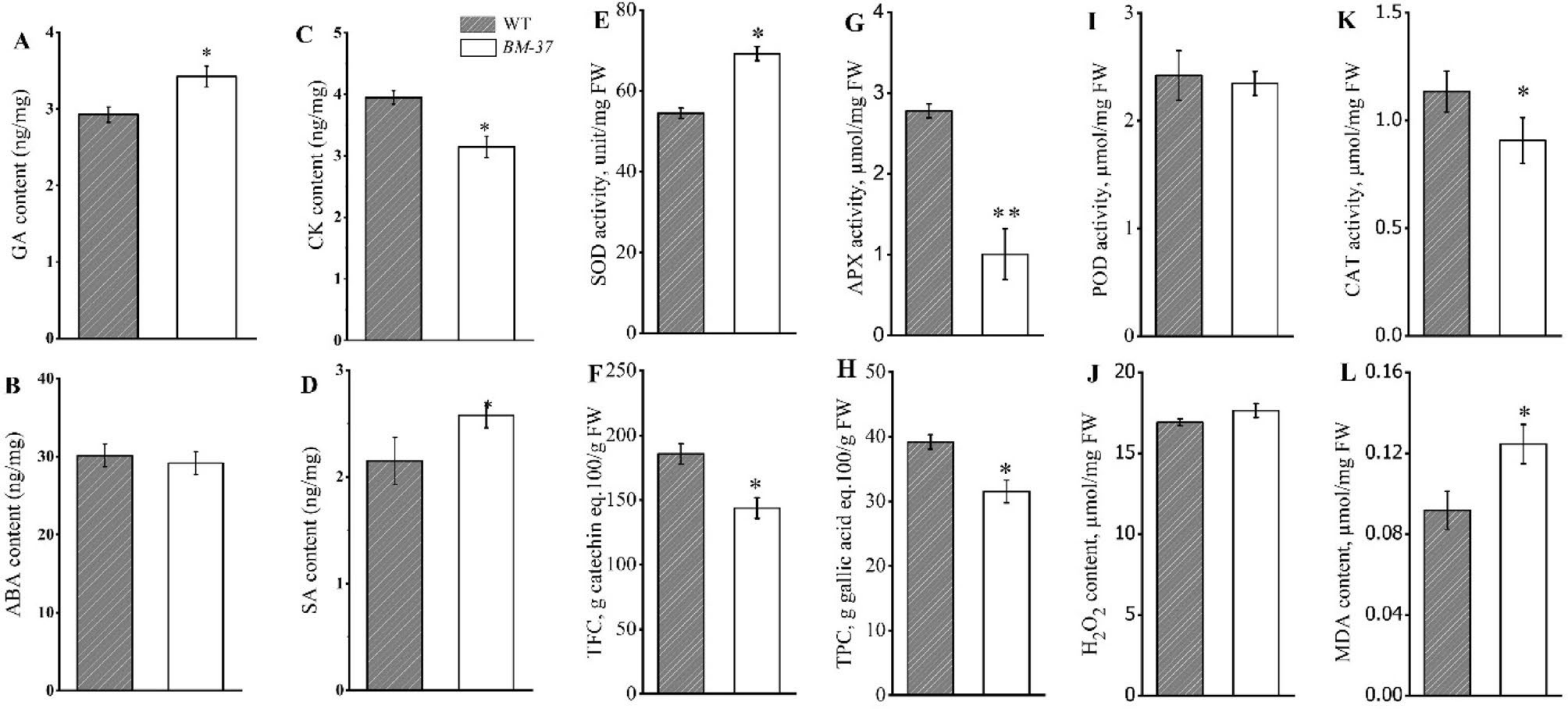
Endegenous phytohormones, enzymatic antioxidants, low molecular weight antioxidants (LMWAs), and reactive oxygen species (ROS) in *BM-37* mutant and WT. (**A**) CK-cytokinins; (**B**) ABA-abscisic acid; (**C**) SA-salicylic acid and (**D**) GA-gibberellic acid; (**E**) SOD-superoxide dismutase; (**G**) APX-ascorbate peroxidase; (**I**) POD-peroxidase; (**K**) CAT-catalase; (**F**) TFC-total flavonoid contents; (**H**) TPC-total phenolics content; (**J**) H_2_O_2_-hydrogen peroxide and (**L**) MDA-malonaldehyde content. All values are the mean ± SD of three biological replications. * p < 0.05 by Tukey’s test.

The *BM-37* mutant and WT leaves at seedling stage were used to assess the levels of the most prevalent growth-related phytohormones, such as SA, CK, GA and ABA in the plant. According to the findings, CK levels were substantially lower and GA and SA contents were significantly higher in the *BM-37* mutant than in the WT, while ABA levels did not differ significantly from those of the WT (Fig. 8A–D). According to the findings, the *BM-37* mutant displayed efficient hormonal signaling for chloroplast formation.

We also evaluated ROS, such as H_2_O_2_ production and MDA contents, and their scavenger antioxidants, such as lower molecular weight antioxidants and enzymatic antioxidants, in order to study the activities of antioxidants for comparison between WT and *BM-37* mutant (Fig. 8E–L). Enzymatic antioxidants like CAT and APX, which are significantly lower in leaves of *BM-37* while SOD is considerably higher in leaves of the WT, might scavenge the oxidative compounds (Fig. 8E, G, K). MDA levels were substantially enhanced in *BM-37* plants than in the WT plants (Fig. 8L), showing that mutant leaves had more oxidative damage. The results also showed that there are no appreciable changes between *BM-37* and WT values for H_2_O_2_ and POD (Fig. 8I, J). The results also depicted that the values of TFC and TPC also significantly reduced in *BM-37* mutant leaves as compared with WT leaves (Fig. 8F, H). The study found that *BM-37* had inadequate antioxidant defense because it was less effective than the WT to scavenge ROS generated by defective chloroplasts.

## Discussion

For gene-tagging, insertional mutagenesis by T-DNA, Tos17 or maize transposons, Ac/Ds and En/dSpm is very useful, but main disadvantage is the frequent presence of somaclonal variation. The frequency of gene tagging by endogenous retrotransposon *Tos17* is very low^47,48^. Mutations by inserting endogenous DNA TEs are desirable to produce transposon-tagged mutant resources. Only the disadvantage with an endogenous DNA TE is to generate stable mutants induced by footprints. In this case, it is difficult to identify the mutant gene. Previously efforts were made to reactivate mPing, a tourist-like thhe tourist-like MITE family member, in the rice genome through tissue culture techniques^49,50^ and gamma-ray irradiations^51–53^. Insertion of mPing induced duplications of targeted site (TSDs) of TTA or TAA, and mPing usually tend to insert into the non-coding region or intron, so mPing was not considered a good transposon-tagging tool. On the other hand, DNA TEs, *nDat1-0* were discovered in rice^9^. Structurally, *nDart1-0* is non-autonomous active DNA transposon with a small size (607-bp), and highly rich GC (about 72%), and tends to transpose into gene regions, it carries a 19-bp perfect segment of terminal inverted repeats (TIRs), which usually has 8-bp high GC targeted site duplications and belongs to *hAT* super family^54,55^. The *nDart1-0* inserted in *OsClpP5* was also found to invade two other genes, indicating its high efficiency for gene-tagging^9^. The blast search revealed that Nipponbare and indica variety 9311 carries 13 and 7 *nDart1* homologs, respectively. The efficient *nDart1-0* utilization for gene-tagging usually depends upon aDart presence or occurrence in that rice variety. It was observed that Nipponbare carries 63 candidates of autonomous element aDart^56^. These specific features distinguish *nDart1-0* from most of the last and previously studies and discoveries of non-autonomous transposable elements in DNA.

The transposable elements caused variegation or albino plants, when these elements expressed in rice varieties^57^. In maize and *Antirrhinum majus*, the transposon tagging genes have been reported, which have been successfully isolated along with different ranges of transposable genes or elements^58–60^. Therefore, we developed a strategy to tag genes mutated by the invasion of *nDart1-0*. The newly developed strategy using iPCR (n1-0SPiPCR) is useful for our *nDart1-0*-specific tagging system. By this system, the transposons elements are usually tagged through an inverse PCR by restriction-ligation-restriction-mediated, which starts from specific sequences of transposon, and amplifies the part of flanking sequences to a specific site for restriction. The resulting PCR products may be used to clone, analyze or sequenced^61–63^. The previous studies elaborated that both aDart and *nDart1-0* have been found useful tool for gene-tagging in japonica and indica rice species. We have found that the utilization of a well-established systematic and computational approach for isolation of active transposon element *nDart1-0* from the host cell genome, may help to understand the pattern of inheritance of transposable elements in rice^64,65^. However, this technique not only the fast method for detecting the *nDart1-0*, while it also has its utilization in gene tagging for various genes in rice genome.

The albino or variegated mutant plants provide important and valuable information regarding plastid growth and development; their genetic analysis has been difficult because of their higher lethal phenotypic effects^66^. From the present study, the muted allele was successfully identified by utilizing the recently developed procedure named as nDart1-0-iPCR. It has been found from tagging for nDart1/aDart1 line that it contained the most frequent and repeated transposable element nDart1-0 while the other line had nDart1 transposable elements^67,68^. It has been found that nDart1 transposable elements showed their behavior to integrate within the promoter regions of genes^69^. Insertion of *nDart1-0* also was found to be a cause for mutation in GTP gene at 13 bp downstream from the initiation ATG codon of GTP gene. There was a frameshift mutation in GTP gene due to *nDart1-0* insertion downstream of GTP gene transcriptional initiation sites. The results from previous research experiments have also revealed that the nDart1 insertion at the transcriptional initiation sites caused effects on the expression levels in downstream gene sites. The *OsClp5* has shown its effects which were disrupted due to insertion on 5’- UTR position of nDart1 transposon in *pyl-v* mutant^70^. It was found that mutant rice line exhibited an increased and enhanced inflorescence, indicating that the *nDart1-0* insertion may cause up-regulated level of gene expression from downstream sites of gene names such as Aberrant Panicle Organization1 in rice^71–73^.

From various research studies, the GTP protein is required in higher amounts for the biogenesis of chloroplast while lower amount for etioplast biogenesis. Translation efficiency of GTP has been found to be activated from etioplast during chloroplast development. The cell division of plastids mostly occurred during P0 up to the very early P4 growth stages, while the activation for photosynthetic apparatus has been found during P4 growth stage of rice liens^74–76^. Our current study suggests that the GTP protein functions during the development of thylakoid membranes of chloroplasts due to a well-established genetic system of plastids. While in the non-green plant tissues, it has also been found that the GTP protein abundance and accumulation during chloroplast development was regulated at the post-transcriptional level.

The *BM-37* mutant, with insertion of *nDart1-0* have lower contents of carotenoid and chlorophyll at seedling stage and a major reduction in pigment would probably affect the chloroplast development and ultimately photosynthesis process (Fig. 6A). Transmission electron microscopy confirmed ultrastructural changes in *BM-37* due to mutation of *nDart1-0* gene (Fig. 5). More number of plastoglobuli observed in *BM-37* that is consistent with the previous research that plastoglobuli number increased when mutants have defect in thylakoid membrane biogenesis and their formation prevent thylakoid from oxidative damage^77^. In *BM-37*, the chloroplast contained smaller starch granule, indicating that starch formation was disturbed, which affect the energy supply for chloroplast development^78^. Moreover, the reduction in chlorophyll contents of *BM-37* mutant have decreased the key photosynthetic parameters, such as the net photosynthetic rate, stomatal conductance, transpiration rate and intercellular CO_2_ concentration (Fig. 6C–F) which was also observed in mutant where gas exchange parameters (*Pn, Tr, g and Ci*) decreased with reduction in chlorophyll contents^79^. In addition, lower transcript level of genes involved in chloroplast development, chlorophyll biosynthesis and photosynthesis of *BM-37* mutant (Fig. 7A–C) have provided the evidence that mutation in *nDart1-0* has disturbed plastid-to-nucleus retrograde signaling which is consistent with the previous finding that chloroplast retrograde signaling was also disrupted in *spc1* mutant of Arabidopsis^80^. Taken together, these results indicated that mutation in *BM-37* was associated with chloroplast development and photosynthetic efficiency.

The *BM-37* mutant plants at seedling stage lead to substantial reduction of chlorophyll and carotenoids contents resulted in impaired chloroplast development that could accumulate higher ROS. Carotenoids provide protection against photo oxidative damage by detoxification of excessive free radicals and reactive oxygen species (ROS)^81^. For instance, impaired chloroplast biogenesis in leaf variegated rice zebra2^25^ as well as mutation in ACD2 encoding red chlorophyll catabolite reductase^82^ resulted in excessive accumulation of ROS. Plant produce anti oxidative enzymes to detoxify ROS such as SOD to cope with superoxides while CAT, POD and APX to scavenge H_2_O_2_. Moreover, it has been reported that non-enzymatic antioxidants can be oxidized to monodehydroascorbate under higher ROS level^83^. Accordingly, our results showed lower contents of non-enzymatic antioxidants (Fig. 8F, H) in *BM-37* mutant plants. Hence, the results inferred that malfunctioning of ROS scavenging enzymes and accumulation of H_2_O_2_ and MDA due to impaired chloroplast development has introduced the yellow leaf color in the *BM-37* mutant at seedling stage.

Under various abiotic stresses, the reactive oxygen species (ROS) and lipid peroxidation produced in crop plants which caused damages in cells, and lead to the death of crop plants. The reduction in growth results from cellular and subcellular abnormalities in plant cells^84–86^. Each crop plant cell has the ability to produce antioxidants (SOD, POD, CAT and APX) to induce the self-defense mechanism in cell, especially for chloroplast and mitochondria^87,88^. The reactive oxygen species (H_2_O_2_) mostly affects the membranes of chloroplast and mitochondria, which ultimately caused death of cell. The production of peroxidase (POD), catalase (CAT), guaiacol peroxidase (GPOD), hydrogen peroxide (H_2_O_2_), superoxide dismutase (SOD), lipoxygenase (LOX), catalase (CAT), ascorbate peroxidase (APOD) and glutathion-S-transferase (GST), glutathione, proline, ascorbic acid, etc. are bioorganic compounds produced in cells^89,90^. The GTP proteins provide an environment for these antioxidants to work under various conditions. The GTP proteins are localized in the cells where there is a need to combat stress conditions. Exploration of various studies on plant hormonal regulation and G-protein signaling reveals several intriguing similarities in their functional roles. In addition, Arabidopsis G-protein mutants display altered phenotypes in response to different plant hormones^91,92^, and transcriptome data reveals a significant change in the expression of G-protein genes under different hormonal treatments^93^.From these studies, it is concluded that transposon nDart1-0 is a useful system to develop mutants for functional gene analysis, and n1-0 SPI PCR is a powerful tool to analyze mutants developed by the insertion of transposon nDart1-0. The transcripts of GTP binding protein were observed in various tissues of plants, usually in the early stages of plants. Era-like GTP binding protein is a new member of an old family, and it is vital for chloroplast biosynthesis and plant survival.

## Conclusions

These studies conclude that transposon *nDart1-0* is a useful system for developinga useful system for developing mutants for gene functional analysis and n1-0 SPI PCR is a powerful tool to analyze mutants developed by the insertion of transposon *nDart1-0*. The transcripts of GTP binding protein were observed in various plant tissues, usually in the early stages of plants. Era-like GTP binding protein is a new member of an old family, and it vital for chloroplast biosynthesis and plant survival. The protocol described may be helpful as a potential method for identifying transposable elements from other organisms, including plants like maize. However, it might have been hoped that this technique described not only the fast method for detecting the *nDart1-0* but also its utilization in gene tagging for various genes in the rice genome.

## Supplementary Information

Supplementary Information.

Supplementary Tables.

Supplementary Figures.

## Data Availability

All of the data generated or analyzed has been provided in the manuscript and supplementary files.

## References

[1] Maurya N, Singh OP, Singh SN, Gautam P, Kumar A (2018). Manuscript effect of temperature on morpho-physiological traits with respect of grain yield of basmati rice. J. Pharmaco. Phytochem..

[2] Yin Y, Wang YF, Cui HL, Zhou R, Li L, Duan GL, Zhu YG (2023). Distinctive structure and assembly of phyllosphere microbial communities between wild and cultivated rice. Microbiol. Spectrum..

[3] Wambugu PW, Ndjiondjop M-N, Henry R (2019). Advances in molecular genetics and genomics of African rice (*Oryza glaberrima* Steud). Plants.

[4] Shaibu AA, Uguru MI, Sow M, Maji AT, Ndjiondjop MN, Venuprasad R (2018). Screening African rice (*Oryza glaberrima*) for tolerance to abiotic stresses: II. Lowland drought. Crop Sci..

[5] Brasileiro AC, Lacorte C, Pereira BM, Oliveira TN, Ferreira DS, Mota AP, Guimaraes PM (2021). Ectopic expression of an expansin-like B gene from wild Arachis enhances tolerance to both abiotic and biotic stresses. The Plant J..

[6] Srivastava S, Pathare VS, Sounderajan S, Suprasanna F (2019). Nitrogen supply influences arsenic accumulation and stress responses of rice (*Oryza sativa* L.) seedlings. J. Hazard. Mater..

[7] International Rice Genome Sequencing Project (2005). The map-based sequence of the rice genome. Nature.

[8] Maekawa M, Tsugane K, Iida S (2011). Effective contribution of the nDart1 transposon-tagging system to rice functional genomics in Advances in Genetics Research (ed. Urbano, K.V.). Nova Sci..

[9] Tsugane K, Maekawa M, Takagi K, Takahara H, Qian Q, Eun CH, Iida S (2006). An active DNA transposon nDart causing leaf variegation and mutable dwarfism and its related elements in rice. Plant J..

[10] Shimatani Z, Takagi K, Eun CH, Maekawa M, Takahara H, Hoshino A, Tsugane K (2009). Characterization of autonomous Dart1 transposons belonging to the hAT superfamily in rice. Mol. Genet. Genom..

[11] Johzuka-Hisatomi, Y. *et al*. Homologous recombination-dependent gene targeting and an active DNA transposon nDart-promoted gene tagging for rice functional genomics. In *Rice Biology in the Genomics Era *81–94 (Springer, 2008).

[12] Ramakrishnan M, Satish L, Sharma A, Kurungara Vinod K, Emamverdian A, Zhou M, Wei Q (2022). Transposable elements in plants: Recent advancements, tools and prospects. Plant Mol. Biol. Rep..

[13] Fang, J. *et al*. A transposon insertion in the OsUBC12 promoter enhances cold tolerance during germination in japonica rice (*Oryza sativa*). 10.21203/rs.3.rs-2541033/v1 (2023).

[14] Takagi K, Maekawa M, Tsugane K, Iida S (2010). Transposition and target preferences of an active nonautonomous DNA transposon nDart1 and its relatives belonging to the hAT superfamily in rice. Mol. Genet. Genom..

[15] Takagi K, Ishikawa N, Maekawa M, Tsugane K, Iida S (2007). Transposon display for active DNA transposons in rice. Genes Genet. Syst..

[16] Hayashi-Tsugane M, Takahara H, Ahmed N, Himi E, Takagi K, Iida S, Maekawa M (2014). A mutable albino allele in rice reveals that formation of thylakoid membranes requires the snow-white leaf1 gene. Plant Cell Physiol..

[17] Zhao Q, Feng Q, Lu H, Li Y, Wang A, Tian Q, Huang X (2018). Pan-genome analysis highlights the extent of genomic variation in cultivated and wild rice. Nat. Genet..

[18] Qiao L, Sinha S, Abd-El-Hafeez AA, Lo IC, Midde KK, Ngo T, Ghosh P (2023). A circuit for secretion-coupled cellular autonomy in multicellular eukaryotic cells. Mol. Syst. Biol..

[19] Jimah JR, Hinshaw JE (2019). Structural insights into the mechanism of dynamin superfamily proteins. Trend Cell Biol..

[20] Meena LS (2011). Cloning and characterization of engA, a GTP-binding protein from *Mycobacterium tuberculosis* H37Rv. Biologicals.

[21] Vernoud V, Horton AC, Yang Z, Nielsen E (2003). Analysis of the small GTPase gene superfamily of Arabidopsis. Plant Physiol..

[22] Shan SO (2016). ATPase and GTPase tangos drive intracellular protein transport. Trends Biochem. Sci..

[23] Kotyada C, Chandra M, Tripathi A, Narooka AR, Verma A (2018). Atypical Switch-I Arginine plays a catalytic role in GTP hydrolysis by Rab21 from Entamoeba histolytica. Biochem. Biophys. Res. Commun..

[24] Doyle JJ (1990). Isolation of plant DNA from fresh tissue. Focus.

[25] Jahan P, Hossain A, Nasiruddin KM, Yasmin S, Khatun F, Parvej MS (2015). mRNA extraction, cDNA synthesis and tillering specific gene isolation from BLB resistant Binashail rice. Asian J. Med. Biol. Res..

[26] Livak KJ, Schmittgen TD (2001). Analysis of relative gene expression data using real-time quantitative PCR and the 2^− ΔΔ^CT method. Methods.

[27] Dellaporta SL, Wood J, Hicks JB (1983). A plant DNA minipreparation: Version II. Plant Mol. Biol. Rep..

[28] Arnon DI (1949). Copper enzymes in isolated chloroplasts. Polyphenoloxidase in Beta vulgaris. Plant Physiol..

[29] Wellburn AR (1994). The spectral determination of chlorophylls a and b, as well as total carotenoids, using various solvents with spectrophotometers of different resolution. J. Plant Physiol..

[30] Singleton VL, Rossi JA (1965). Colorimetry of total phenolics with phosphomolybdic-phosphotungstic acid reagents. Am. J. Enol. Vitic..

[31] Zhishen J, Mengcheng T, Jianming W (1999). The determination of flavonoid contents in mulberry and their scavenging effects on superoxide radicals. Food Chem..

[32] Zhang WF, Zhang F, Raziuddin R, Gong HJ, Yang ZM, Lu L (2008). Effects of 5-aminolevulinic acid on oilseed rape seedling growth under herbicide toxicity stress. J. Plant Growth Regul..

[33] Zhou W, Leul M (1999). Uniconazole-induced tolerance of rape plants to heat stress in relation to changes in hormonal levels, enzyme activities and lipid peroxidation. Plant Growth Regul..

[34] Aebi H (1984). Catalase in vitro. Meth. Enzymol..

[35] Nakano Y, Asada K (1981). Hydrogen peroxide is scavenged by ascorbate-specific peroxidase in spinach chloroplasts. Plant Cell Physiol..

[36] Velikova V, Yordanov I, Edreva A (2000). Oxidative stress and some antioxidant systems in acid rain-treated bean plants: Protective role of exogenous polyamines. Plant Sci..

[37] Morales M, Munné-Bosch S (2019). Malondialdehyde: Facts and artifacts. Plant Physiol..

[38] Zhishen J, Mengcheng T, Jianming W (1999). The determination of flavonoid contents in mulberry and their scavenging effects on superoxide radicals. Food Chem..

[39] Wu Z, Zhang X, He B, Diao L, Sheng S, Wang J, Wan J (2007). A chlorophyll-deficient rice mutant with impaired chlorophyllide esterification in chlorophyll biosynthesis. Plant Physiol..

[40] Kyozuka J, McElroy D, Hayakawa T, Xie Y, Wu R, Shimamoto K (1993). Light-regulated and cell-specific expression of tomato rbcS-gusA and rice rbcS-gusA fusion genes in transgenic rice. Plant Physiol..

[41] Kusumi K, Sakata C, Nakamura T, Kawasaki S, Yoshimura A, Iba K (2011). A plastid protein NUS1 is essential for build-up of the genetic system for early chloroplast development under cold stress conditions. Plant J..

[42] Takeuchi R, Kimura S, Saotome A, Sakaguchi K (2007). Biochemical properties of a plastidial DNA polymerase of rice. Plant Mol. Biol..

[43] Vitha S, McAndrew RS, Osteryoung KW (2001). FtsZ ring formation at the chloroplast division site in plants. J. Cell Biol..

[44] Yoo SC, Cho SH, Sugimoto H, Li J, Kusumi K, Koh HJ, Paek NC (2009). Rice virescent3 and stripe1 encoding the large and small subunits of ribonucleotide reductase are required for chloroplast biogenesis during early leaf development. Plant Physiol..

[45] Gong X, Jiang Q, Xu J, Zhang J, Teng S, Lin D, Dong Y (2013). Disruption of the rice plastid ribosomal protein S20 leads to chloroplast developmental defects and seedling lethality. G3 Genes Genom. Genet..

[46] Hiratsuka J, Shimada H, Whittier R, Ishibashi T, Sakamoto M, Mori M, Sugiura M (1989). The complete sequence of the rice (*Oryza sativa*) chloroplast genome: Intermolecular recombination between distinct tRNA genes accounts for a major plastid DNA inversion during the evolution of the cereals. Mol. Gen. Genet..

[47] Hirochika H (2001). Contribution of the Tos17 retrotransposon to rice functional genomics. Curr. Opin. Plant Biol..

[48] Hirochika H, Guiderdoni E, An G, Hsing YI, Eun MY, Han CD, Leung H (2004). Rice mutant resources for gene discovery. Plant Mol. Biol..

[49] Kikuchi K, Terauchi K, Wada M, Hirano HY (2003). The plant MITE mPing is mobilized in anther culture. Nature.

[50] Liu D, Zhang S, Fauquet C, Crawford NM (1999). The Arabidopsis transposon Tag1 is active in rice, undergoing germinal transposition and restricted, late somatic excision. Mol. Gen. Genet..

[51] Goff SA (1999). Rice as a model for cereal genomics. Curr. Opin. Plant Biol..

[52] Jiang N, Bao Z, Zhang X, Hirochika H, Eddy SR, McCouch SR, Wessler SR (2003). An active DNA transposon family in rice. Nature.

[53] Nakazaki T, Okumoto Y, Horibata A, Yamahira S, Teraishi M, Nishida H, Tanisaka T (2003). Mobilization of a transposon in the rice genome. Nature.

[54] Kumar A, Hirochika H (2001). Applications of retrotransposons as genetic tools in plant biology. Trend. Plant Sci..

[55] Koonin E, Galperin MY (2002). Sequence-Evolution-Function: Computational Approaches in Comparative Genomics.

[56] Komatsu M, Shimamoto K, Kyozuka J (2003). Two-step regulation and continuous retrotransposition of the rice LINE-type retrotransposon Karma. Plant Cell.

[57] Huang Y, Shukla H, Lee YCG (2022). Species-specific chromatin landscape determines how transposable elements shape genome evolution. Elife.

[58] Khush GS (1997). Origin, dispersal, cultivation and variation of rice. Plant Mol. Biol..

[59] Lin X, Long L, Shan X, Zhang S, Shen S, Liu B (2006). In planta mobilization of mPing and its putative autonomous element Pong in rice by hydrostatic pressurization. J. Exp. Bot..

[60] Walbot V (2000). Saturation mutagenesis using maize transposons. Curr. Opin. Plant Biol..

[61] Harris SB (2002). Virtual rice. EMBO Rep..

[62] Hamer L, DeZwaan TM, Montenegro-Chamorro MV, Frank SA, Hamer JE (2001). Recent advances in large-scale transposon mutagenesis. Curr. Opin. Chem. Biol..

[63] Kempin SA, Liljegren SJ, Block LM, Rounsley SD, Yanofsky MF, Lam E (1997). Targeted disruption in Arabidopsis. Nature.

[64] Goff SA, Ricke D, Lan TH, Presting G, Wang R, Dunn M, Briggs S (2002). A draft sequence of the rice genome (*Oryza sativa* L. ssp. japonica). Science.

[65] Gale MD, Devos KM (1998). Comparative genetics in the grasses. Proc. Natl. Acad. Sci..

[66] Yang S, Overlander M, Fiedler J (2021). Genetic analysis of the barley variegation mutant, grandpa1. BMC Plant Biol..

[67] Miyao A, Tanaka K, Murata K, Sawaki H, Takeda S, Abe K, Hirochika H (2003). Target site specificity of the Tos17 retrotransposon shows a preference for insertion within genes and against insertion in retrotransposon-rich regions of the genome. Plant Cell.

[68] Takagi K, Ishikawa N, Maekawa M, Tsugane K, Iida S (2007). Transposon display for active DNA transposons in rice. Gene. Genet. Syst..

[69] May BP, Martienssen RA (2003). Transposon mutagenesis in the study of plant development. Crit. Rev. Plant Sci..

[70] Sato Y, Sentoku N, Miura Y, Hirochika H, Kitano H, Matsuoka M (1999). Loss-of-function mutations in the rice homeobox gene OSH15 affect the architecture of internodes resulting in dwarf plants. EMBO J..

[71] Sasaki T, Burr B (2000). International Rice Genome Sequencing Project: The effort to completely sequence the rice genome. Curr. Opin. Plant Biol..

[72] Murai N, Li Z, Kawagoe Y, Hayashimoto A (1991). Transposition of the maize activator element in transgenic rice plants. Nucleic Acids Res..

[73] Takano M, Kanegae H, Shinomura T, Miyao A, Hirochika H, Furuya M (2001). Isolation and characterization of rice phytochrome A mutants. Plant Cell.

[74] Agrawal GK, Yamazaki M, Kobayashi M, Hirochika R, Miyao A, Hirochika H (2001). Screening of the rice viviparous mutants generated by endogenous retrotransposon Tos17 insertion. Tagging of a zeaxanthin epoxidase gene and a novel OsTATC gene. Plant Physiol..

[75] Tyagi AK, Mohanty A (2000). Rice transformation for crop improvement and functional genomics. Plant Sci..

[76] Yu J, Hu S, Wang J, Wong GKS, Li S, Liu B, Yang H (2002). A draft sequence of the rice genome (*Oryza sativa* L. ssp. indica). Science.

[77] Bédard J, Trösch R, Wu F, Ling Q, Flores-Pérez Ú, Töpel M, Nawaz F, Jarvis P (2017). Suppressors of the chloroplast protein import mutant reveal a genetic link between protein import and thylakoid biogenesis. Plant Cell.

[78] Deng L, Qin P, Liu Z, Wang G, Chen W, Tong J, Xiao L, Tu B, Sun Y, Yan W, He H, Tan J, Chen X, Wang Y, Li S (2017). Characterization and fine-mapping of a novel premature leaf senescence mutant yellow leaf and dwarf 1 in rice. Plant Physiol. Biochem..

[79] Gang H, Li R, Zhao Y, Liu G, Chen S, Jiang J (2019). Loss of GLK1 transcription factor function reveals new insights in chlorophyll biosynthesis and chloroplast development. J. Exp. Bot..

[80] Dong H, Deng Y, Mu J, Lu Q, Wang Y, Xu Y, Chu C, Chong K, Lu C, Zuo J (2007). The Arabidopsis spontaneous cell death1 gene, encoding a ζ-carotene desaturase essential for carotenoid biosynthesis, is involved in chloroplast development, photoprotection and retrograde signaling. Cell Res..

[81] Howitt CA, Pogson BJ (2006). Carotenoid accumulation and function in seeds and non-green tissues. Plant Cell Environ..

[82] Yao N, Greenberg JT (2006). Arabidopsis accelerated cell death2 modulates programmed cell death. Plant Cell.

[83] Apel K, Hirt H (2004). Reactive oxygen species: Metabolism, oxidative stress, and signal transduction. Annu. Rev. Plant Biol..

[84] Jan S, Noman A, Kaya C, Ashraf M, Alyemeni MN, Ahmad P (2020). 24-Epibrassinolide alleviates the injurious effects of Cr (VI) toxicity in tomato plants: Insights into growth, physio-biochemical attributes, antioxidant activity and regulation of Ascorbate–glutathione and Glyoxalase cycles. J. Plant. Growth Regul..

[85] Ahmad R, Ali S, Rizwan M, Dawood M, Farid M, Hussain A, Wijaya L, Alyemeni MN, Ahmad P (2020). Hydrogen sulfide alleviates chromium stress on cauliflower by restricting its uptake and enhancing antioxidative system. Physiol. Plant.

[86] Lei K, Sun S, Zhong K, Li S, Hu H, Sun C, Zheng Q, Tian Z, Dai T, Sun J (2021). Seed soaking with melatonin promotes seed germination under chromium stress via enhancing reserve mobilization and antioxidant metabolism in wheat. Ecotoxicol. Environ. Saf..

[87] Wakeel A, Xu M, Gan Y (2020). Chromium-induced reactive oxygen species accumulation by altering the enzymatic antioxidant system and associated cytotoxic, genotoxic, ultrastructural, and photosynthetic changes in plants. Int. J. Mol. Sci..

[88] Fan W-J, Feng Y-X, Li Y-H, Lin Y-J, Yu X-Z (2020). Unraveling genes promoting ROS metabolism in subcellular organelles of Oryza sativa in response to trivalent and hexavalent chromium. Sci. Total Environ..

[89] Wang M, Zhang S, Ding F (2020). Melatonin mitigates chilling-induced oxidative stress and photosynthesis inhibition in tomato plants. Antioxidants.

[90] Dreyer A, Dietz K-J (2018). Reactive oxygen species and the redox-regulatory network in cold stress acclimation. Antioxidants..

[91] Xu DB, Chen M, Ma YN, Xu ZS, Li LC, Chen YF, Ma YZ (2015). A G-protein β subunit, AGB1, negatively regulates the ABA response and drought tolerance by down-regulating AtMPK6-related pathway in Arabidopsis. PLoS ONE.

[92] Roy Choudhury S, Li M, Lee V, Nandety RS, Mysore KS, Pandey S (2020). Flexible functional interactions between G-protein subunits contribute to the specificity of plant responses. Plant J..

[93] Jose J, Choudhury SR (2020). Heterotrimeric G-proteins mediated hormonal responses in plants. Cell. Signal..

