# Supplementary material for: Molecular and biochemical characterization of rice developed through conventional integration of nDart1-0 transposon gene
Source: Sci Rep. 2023 May 19;13:8139. doi: 10.1038/s41598-023-35095-7 (PMC10199049; doi:10.1038/s41598-023-35095-7)
Supplement: Supplementary file 1 — Supplementary Information. [file 41598_2023_35095_MOESM1_ESM.docx]

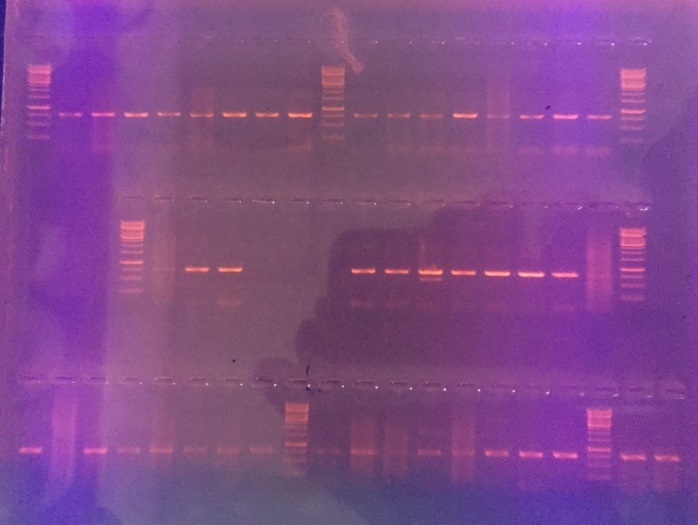


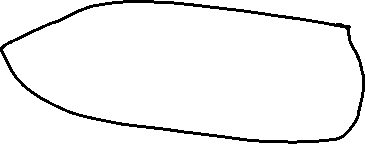


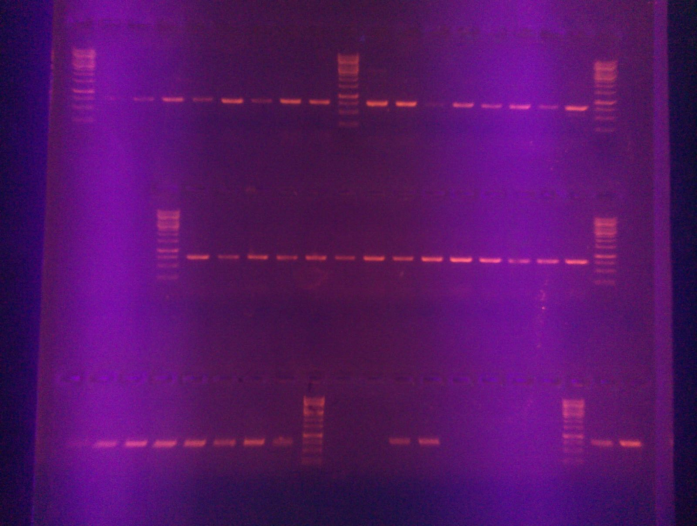
A.


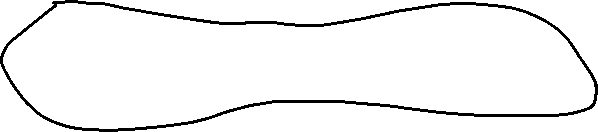


B

**Figure 3:** PCR amplification of nDart1-0 mutants and transcripts of GTP binding protein gene in several tissues of Basmati 370. (A) PCR amplification of nDart1-0 mutants. Lane 1: Basmati 370, Lane2: T-65, Lane3: Nipponbare, Lane4: Mutable whitish-leaf, Lane 5: Stable whitish leaf-1, Lane6: Stable whitish leaf-2, Lane7: Stable whitish leaf-3; (B) Transcripts of GTP binding protein gene in several tissues of Basmati 370. Lane 1: Etiolated plant leaf, Lane2: Etiloated plant root, Lane 3: 2-week old plant leaf, Lane 4: 2-week old plant root, Lane5: 2-Month plant leaf, Lane6: 2-Month old plant root, Lane 7: 2Month plant stem, Lane8: 2-Month old plant meristem, Lane9: 2-Month old plant glume, Lane 10: 2-Month old plant anther, Lane11-2Month old plant stigma
