# Supplementary material for: Molecular and biochemical characterization of rice developed through conventional integration of nDart1-0 transposon gene
Source: Sci Rep. 2023 May 19;13:8139. doi: 10.1038/s41598-023-35095-7 (PMC10199049; doi:10.1038/s41598-023-35095-7)
Supplement: Supplementary file 2 — Supplementary Tables. [file 41598_2023_35095_MOESM2_ESM.docx]

**Supplementary Tables**

**Supplementary Table 1**. List of oligonucleotide primers for full length cDNA amplification

| **Oligonucleotide Primers** | | |
| --- | --- | --- |
| Forward Primers | GB-1F | 5' GGCGGTATCATCTCGCATTTCAC 3' |
|  | GB-2F | 5' CTCCCAAGCCGAGGAGCTCTCG 3' |
| Reverse Primers | GB-1R | 5' GCCAACGGCACTCCTAACATTCT 3' |
|  | GB-2R | 5' TAACCGCTGCGGTGGTTGGCAC 3' |

**Supplementary Table 2**. Markers designed for qRT-PCR

| **Gene** | **Discription** | **Forward primer** | **Reverse primer** |
| --- | --- | --- | --- |
| *Rps7* | encoding ribosomal proteins S7 | gccaaaatccattccaattc | ggagatgtacacgaggagattg |
| *Rps20* | encoding ribosomal proteins S20 | CACGCTCTTCTCCCTCTCCT' | GTAGGAGGCGGACAGGCG |
| *Cab 1R* | encoding chlorophyll binding protein PSII | CCGTTGGTGTTCTTTCCGAT | TTCAAGTCCGCCGTCAAATC |
| *Rpl21* | encoding the ribosomal protein L21 | AAGAAGAGGAGGCTGCGGT | GACATTGGCGCCTTTCAGC |
| *RNRL* | encoding large subunits of ribonucleotide reductase | gttagatgcttcactacacag | gtaccattgccaacatggcaac |
| *RNRS* | encoding small subunits of ribonucleotide reductase | gccaaaatccattccaattc | ggagatgtacacgaggagattg |
| *LhcpII* | encoding light harvesting complex II of photosystem | GAAGAAGATCAAGAACGGCC | TTGCCGGGGACGAAGTTGGT |
| *RbcS* | encoding small subunit of Rubisco | TCCGCTGAGTTTTGGCTATTT | GGACTTGAGCCCTGGAAGG |
| *FtsZ* | encoding component of plastid division machinery | AAAGGACATAACCTTGCAAG | AGTTTTCCTATTGAACCGTG |
| *OsPOLP1* | encoding 1 plastidial DNA polymerase | ACCGGTGCTTTCAGGCTTGG | GCTGACTGATAATCACACG |
| *OsRpoTp* | encoding NEP core subunits | AAGCAGACAGTGATGACATC | ATCACATGCATGCACCCAAA |
| *RpoB* | encoding PEP core b subunits | TTTGGTTTCGATGTGCA | TATGGTCTAATTCCGAGCGGT |
| *PsaA* | encoding two reaction center polypeptides | GCGAGCAAATAAAACACCTTTC | GTACCAGCTTAACGTGGGGAG |
| *PsbA* | encoding two reaction center polypeptides | CCCTCATTAGCAGATTCGTTTT | ATGATTGTATTCCAGGCAGAGC |
| *RbcL* | encoding large subunit of Rubisco | CTTGGCAGCATTCCGAGTAA | ACAACGGGCTCGATGTGATA |
| *RpoA* | encoding PEP core a subunits | GTGGAAGTGTGTTGAATCAA | TCTCTCTTGATCCGTAACTC |
| *CAO1* | Chlorophyllide a oxygenase1 | GATCCATACCCGATCGACAT | CGAGAGACATCCGGTAGAGC |
| *YGL1* | encoding a chlorophyll synthetase | CAGTCTCCAATGGCCACCT | TGCTTTCATCAGTGGCTGGT |
| *HEMA* | encoding glutamyl tRNA reductase | AGATGGGTTTAGTGCGACGAG | TTTGGGATCGAGGGAGTATTT |
| *CHLD* | encoding Mg-chelatase D subunit | AGGAAGGCTGGAAGAGGACC | CGGGAAATTGTGAGGGACAT |
| *PORA* | encoding NADPH dependent protochlorophyllide oxidoreductase | TGTACTGGAGCTGGAACAACAA | GAGCACAGCAAAATCCTAGACG |

**Supplementary Table 3.** Analysis of the mutants tagged by the insertion of transposon *nDart1-0*

| **Clone region** | **Chr. No** | **Gene** | **Flanking** | **Inserted** |
| --- | --- | --- | --- | --- |
| OJ1793_E11 | 7 | ATP-dependent | 56580 | 2nd exon |
| OSJNBb0089K24 | 4 | DNA polymerase (F) | 77444 | 6th intron |
| OSJNBa0024J08 | 12 | GA receptor | 31292 | Ist exon |
| OSJNBb0024B16 | 3 | GNS1/SUR4 | 114222 | Promoter (-163) |
| OSJNBa0049D13 | 5 | Glycosyl transferase | 79883 | 8th intron |
| OJ1342_D02 | 2 | Reductase | 5231 | Promoter(-283 |
| OSJNBa0050A24 | 5 | Unknown | 107816 | 2nd exon |
| OJ1118_F06 | 5 | ATPase dependent | 44538 | 6th intron |
| P0047B07 | 7 | Sec 1P | 59433 | Promoter (-158) |
| OSJNBa0010K05 | 11 | Ankyrin repeat | 91478 | Promoter (-559) |
| OSJNBa0043A12 | 4 | RecX family | 36761 | Promoter (-170) |
| B1154F06 | 3 | Unknown | 13884 | 2nd exon |
| P0038F12 | 1 | Argonaut2 | 76409-76255 | Ist exon |
| P0436F11 | 6 | RRM- protein | 136607-136780 | 2nd intron |
| P0030G11 | 2 | cyclin-dependent | 55714-55698 | Promote (-224) |
| P0499C11 | 1 | RNA binding | 94767-94821 | Promoter (-126) |
| B1469H02 | 2 | auxin response factor | 52156-52327 | Promoter (-155) |
