# Supplementary material for: Molecular and biochemical characterization of rice developed through conventional integration of nDart1-0 transposon gene
Source: Sci Rep. 2023 May 19;13:8139. doi: 10.1038/s41598-023-35095-7 (PMC10199049; doi:10.1038/s41598-023-35095-7)
Supplement: Supplementary file 3 — Supplementary Figures. [file 41598_2023_35095_MOESM3_ESM.docx]

**Supplementary Figures**

**Figure-S1**


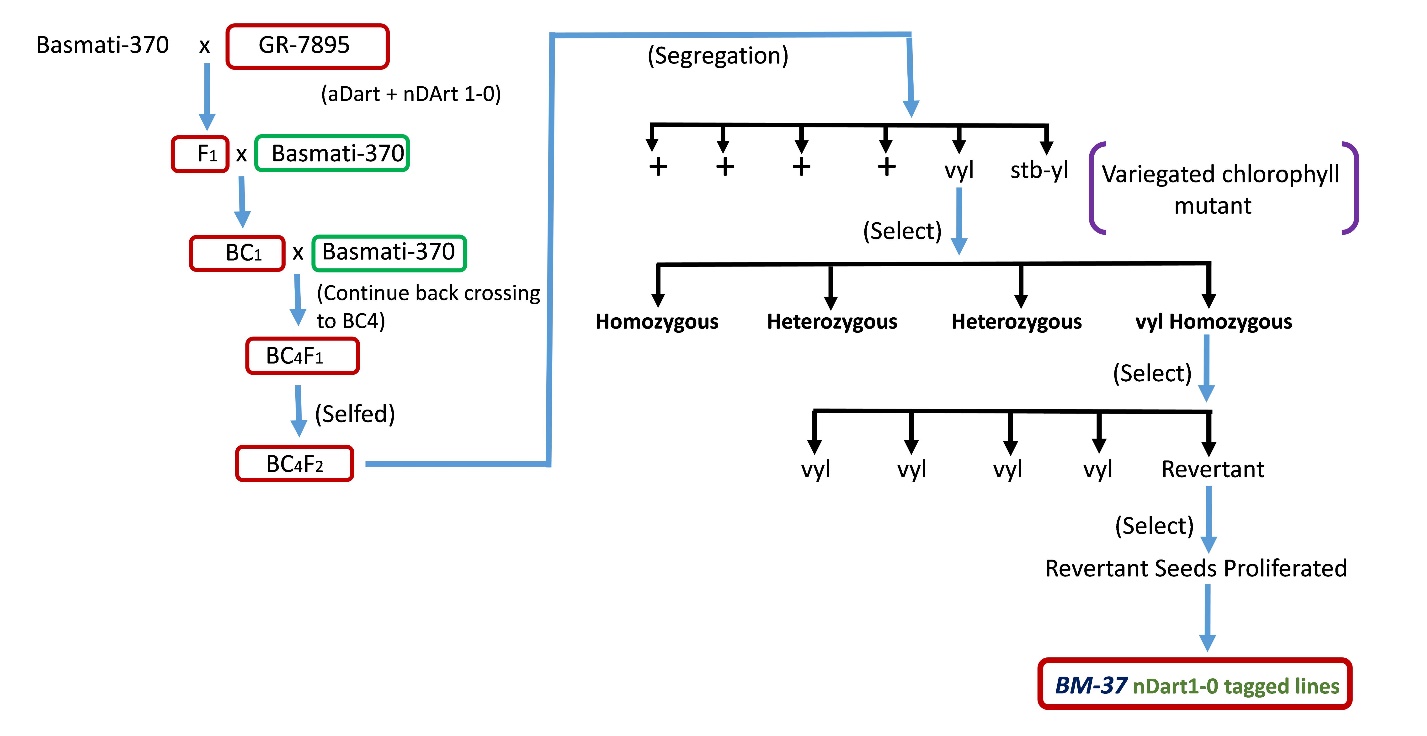


**Supplementary Figure 1:** Layout diagram of conventionally transformation of *nDart1-0* breeding programme for *BM-37* mutant development

**Figure-S2**

**HOLD 1**

**2 HOLD**

**3 Temp 35 Cycles**

3:00

0:30

0:30

1:00

10:00

∞

60.0

95.0

95.0

72.0

72.0

4.0

**Supplementary Figure 2:** PCR conditions for gene amplification

**Figure-S3**


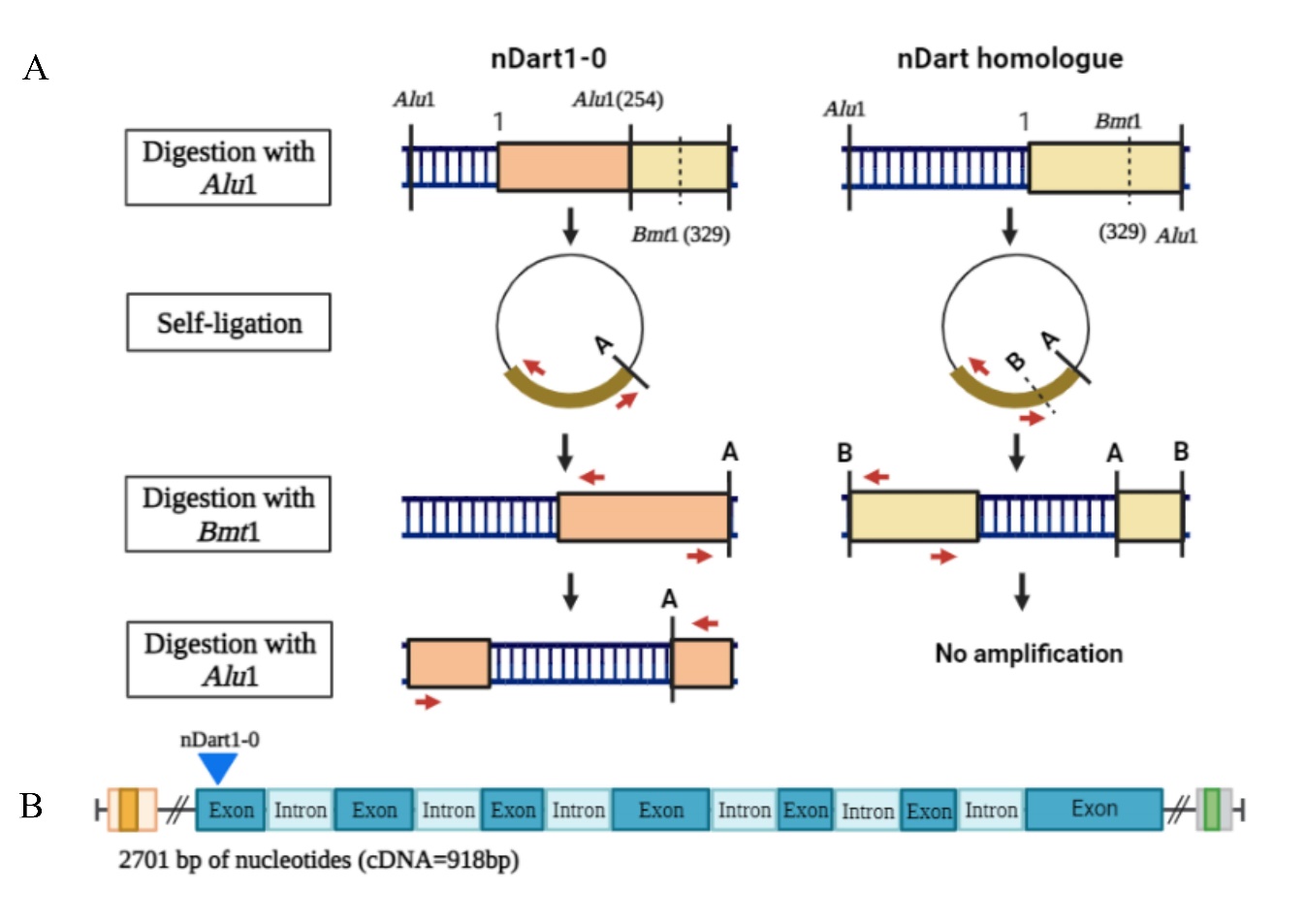


**Supplementary Figure 3:** (A) nDart1-O Specific inverse PCE (n1-OSPiPCR); (B) GTP-binding protein locus on the BAC clone OJ1781_H11 of chromosome 5 was found to contain an insertion of DNA transposon nDart1-0.
